# Columnar/herringbone dual crystal packing of pyrenylsumanene and its photophysical properties

**DOI:** 10.3762/bjoc.10.80

**Published:** 2014-04-11

**Authors:** Binod Babu Shrestha, Shuhei Higashibayashi, Hidehiro Sakurai

**Affiliations:** 1 Department of Functional Molecular Science, School of Physical Sciences, The Graduate University for Advanced Studies, Myodaiji, Okazaki, Aichi 444-8787, Japan; 2 Research Center of Integrative Molecular Systems, Institute for Molecular Science, Myodaiji, Okazaki, Aichi 444-8787, Japan,; 3 Japan Science and Technology Agency, ACT-C, 4-1-8 Honcho, Kawaguchi, Saitama 332-0012, Japan

**Keywords:** carbon nanomaterials, columnar crystal packing, fluorescence, herringbone, pyrenylsumanene

## Abstract

A single crystal of pyrenylsumanene was found to exhibit both columnar and herringbone crystal packing. The sumanene moieties form unidirectional columnar structures based on π–π stacking while the pyrene moieties generate herringbone structures due to CH–π interactions. The absorption and emission maxima of pyrenylsumanene were both red-shifted relative to those of sumanene and pyrene, owing to the extension of π-conjugation. Monomer emission with high quantum yield (0.82) was observed for pyrenylsumanene in solution, while excimer-type red-shifted emission was evident in the crystalline phase.

## Introduction

**Figure 1:**
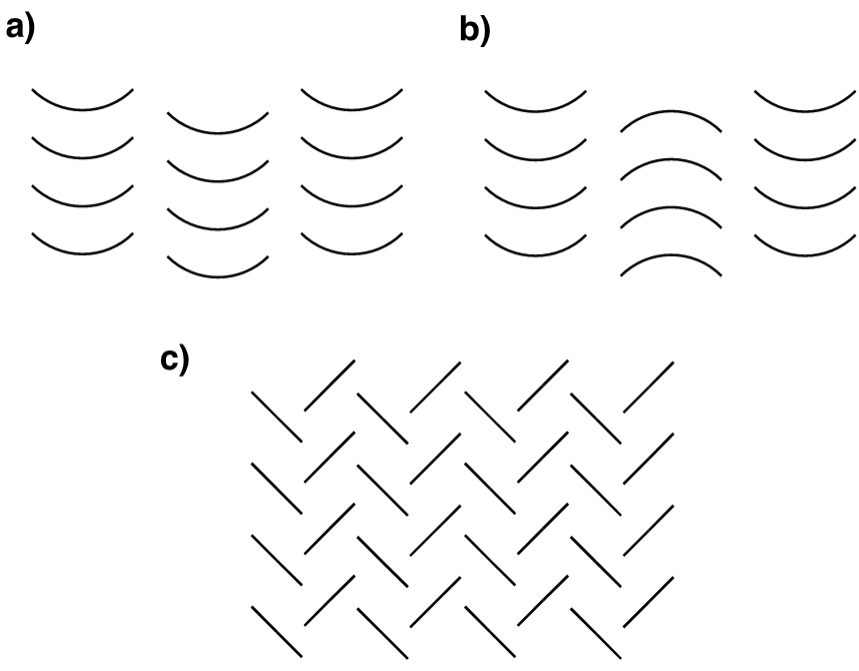
Columnar packing structures of buckybowls showing (a) unidirectional and (b) opposite structures, in addition to (c) the herringbone packing structure typical of planar π-conjugated compounds.

Buckybowls – bowl-shaped aromatic hydrocarbons – possess unique physical properties due to their curved π-conjugated systems [1–5]. One of these characteristic features is a columnar packing structure in the crystal state. Many buckybowls, including sumanene [2], exhibit columnar packing in which the bowl-shaped molecules are stacked in a convex-to-concave fashion, since this particular pattern results in more highly favored intermolecular π–π interactions [1–13]. The columnar packing structures of buckybowls typically occur in two forms which are differentiated by the stacking of the columns: unidirectional (Figure 1a) and opposite (Figure 1b). These columnar structures allow buckybowls to exhibit specific solid-state properties, including high electron conductivity and solid-state emission [14–18]. In contrast, planar π-conjugated aromatic compounds tend to favour a herringbone packing structure (Figure 1c) due to π–π and CH–π interactions [19–20]. To date, buckybowl derivatives with planar aromatic substituents have not been well studied and thus we wished to examine the crystal packing modes and solid-state properties of dual-nature compounds incorporating both bowl and planar structures. Pyrene was selected as the planar substituent when studying solid-state photophysical properties [21–22] and herein we report the columnar/herringbone dual crystal packing of pyrenylsumanene (**1**) in addition to its photophysical properties.

## Results and Discussion

**Scheme 1:**
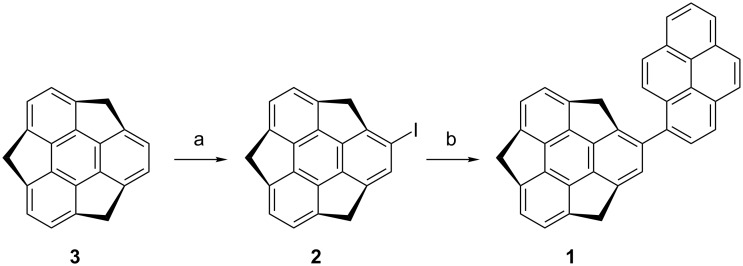
Synthesis of pyrenylsumanene (**1**): (a) Sc(OTf)_3_ (5 mol %), DIH (100 mol %), CH_2_Cl_2_, rt, 2.5 h, yield 80%; (b) palladium (II) acetate (20 mol %), 1-pyreneboronic acid (150 mol %), acetone/water 4:1, 40 °C, 12 h, yield 84%.

Pyrenylsumanene (**1**) was prepared from iodosumanene [8] and pyreneboronic acid in 84% yield through a Suzuki–Miyaura cross-coupling reaction (Scheme 1). The preparation of iodosumanene was improved by using a catalytic amount of scandium (III)triflate (Sc(OTf)_3_) with 6,6’-diiodo-2,2’-dimethoxy-1,1’-binaphthol (DIH) [23] compared to the previously reported system of AuCl_3_ and *N*-iodosuccinimide [8], resulting in an 80% yield.

**Figure 2:**
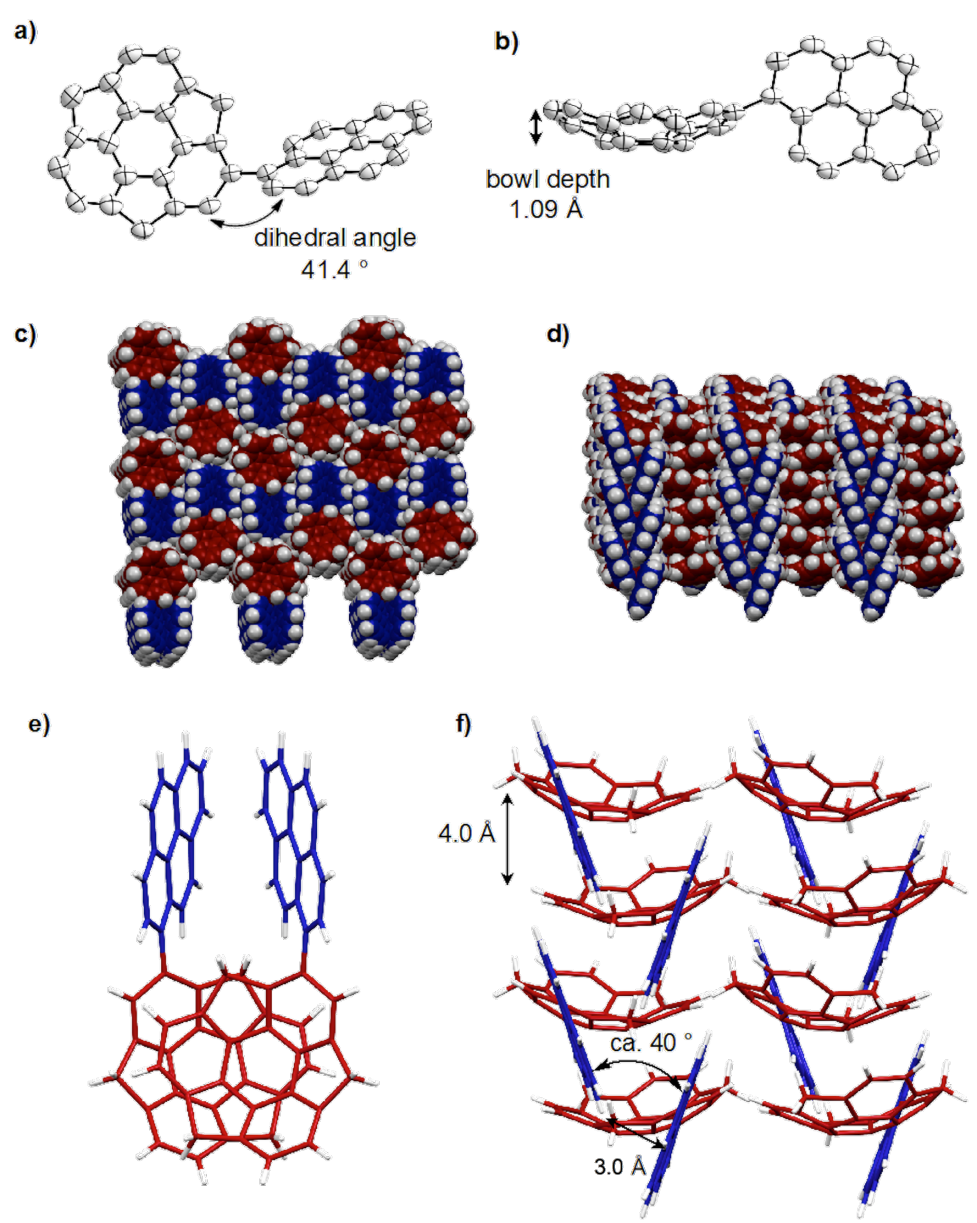
The X-ray crystal structure of **1**, showing: (a) top view of the ORTEP drawing with 50% probability, b) side view, c) top view of the packing structure with the sumanene bowl in red and the pyrene substituent in blue, d) side view, e) top view of column stacking of enantiomers and f) side view of columns with a herringbone packing of the pyrene moiety due to CH–π interactions.

Following synthesis of **1**, a single crystal was obtained from CH_2_Cl_2_/MeOH solution, with the crystal structure shown in Figure 2. The bowl depth of **1** from the centroid of the rim carbons to the centroid of the benzene ring is 1.09 Å (Figure 2b) and thus the bowl is slightly shallower than that of sumanene (1.11 Å) [11]. The structure resulting from DFT calculations at the ωB97XD/6-31G(d) level indicates that **1** has a greater bowl depth (1.15 Å) than sumanene (1.13 Å). This difference results from the effects of intermolecular interactions in the crystal state [9]. The X-ray data indicate that the dihedral angle between the sumanene and pyrene moieties is 41.4° (Figure 2a). Most notably, **1** exhibits dual columnar and herringbone packing modes; the sumanene moiety undergoes columnar packing with convex-to-concave stacking, while the pyrene moiety shows herringbone packing with CH–π interactions (Figures 2c,d). The columns of **1** are arranged unidirectionally, in the same manner as observed in sumanene and hexafluorosumanene (representing the type a stacking shown in Figure 1) [7,11]. Compound **1** possesses bowl chirality [9,24–25] and the crystal represents a racemic mixture in which the two enantiomers are stacked in columns alternating at 4.0 Å intervals with side-to-side offsets (Figures 2e,f). In the herringbone arrangement of the pyrene moieties, the CH–π interactions occur at a distance of 3.0 Å (Figure 1f). The π–π stacking of pyrene moieties, however, is not evident in the arrangement.

**Figure 3:**
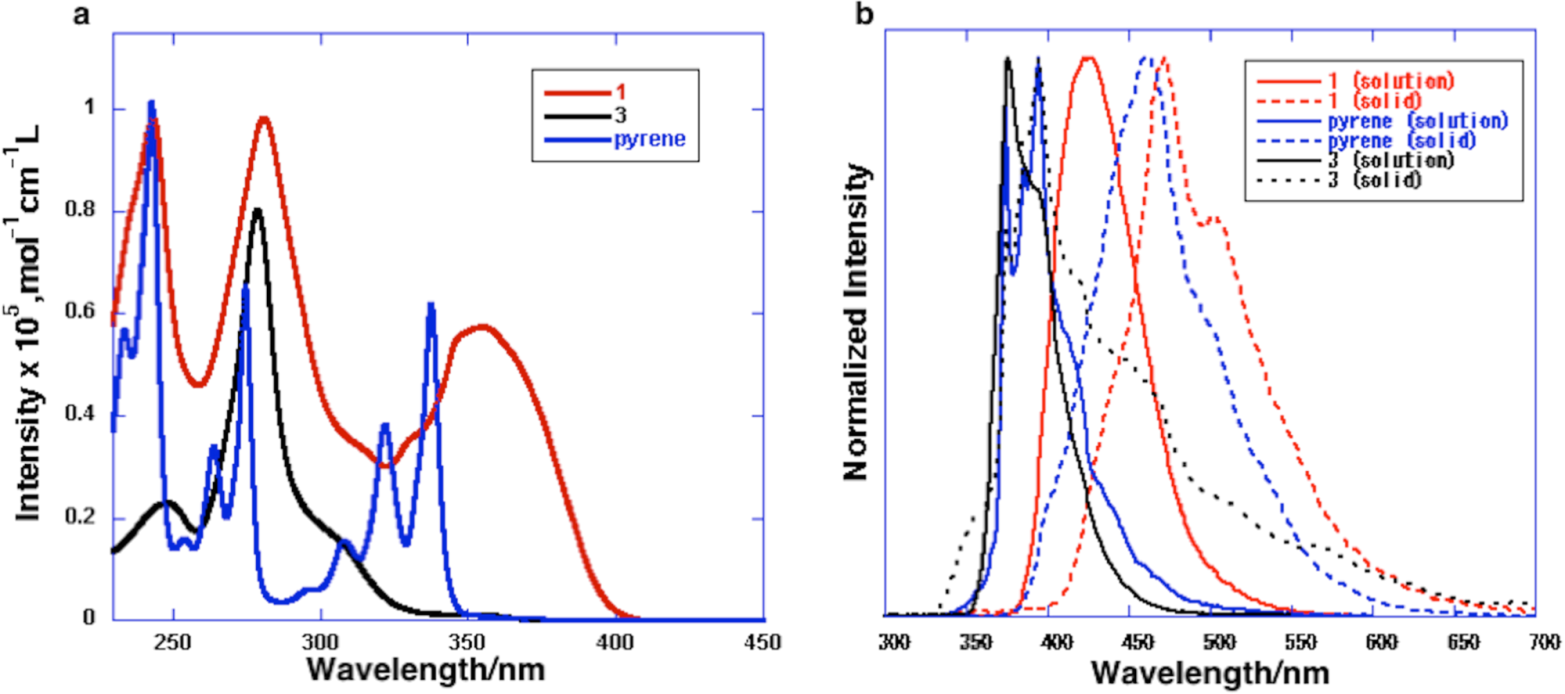
(a) Absorption spectra of **1**, **3** and pyrene in CH_2_Cl_2_ solution (1.0 × 10^−5^ M); (b) emission spectra of **1**, **3** and pyrene in CH_2_Cl_2_ solution (solid line) (1.0 × 10^−5^ M) and in the solid state (dotted line).

**Figure 4:**
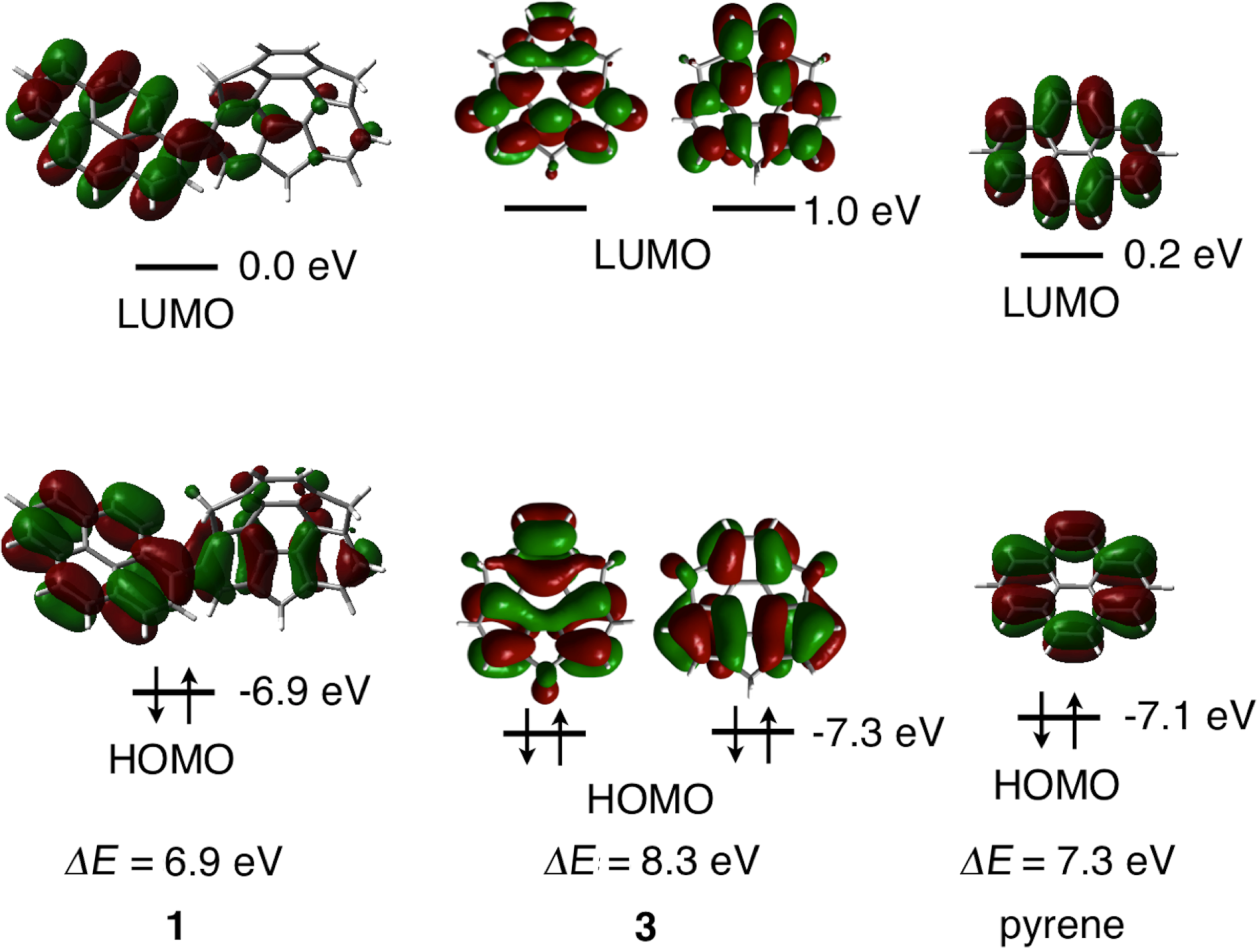
Calculated HOMO and LUMO orbitals and HOMO–LUMO gaps (ΔE) for **1**, **3** and pyrene (ωB97XD/6-31G(d)).

The UV–vis absorption and emission spectra and maxima as well as the quantum yields of **1**, **3** and pyrene in CH_2_Cl_2_ or in the solid state are summarized in Figure 3 and Table 1. The absorption maximum of **1** was evidently red-shifted relative to those of **3** and pyrene. The 355 nm absorption band of **1** was assigned to the HOMO–LUMO transition by TD-DFT calculations (ωB97XD/6-31G(d)). DFT calculations also demonstrated that the HOMO and LUMO of **1** are primarily located on the pyrene moiety (Figure 4). The dihedral angle between the sumanene and pyrene moieties resulting from calculations was 48.2°. This angle causes some extension of the π-conjugation to the sumanene moiety, resulting in a narrower HOMO–LUMO gap and the observed red shift in absorption. The emission of **1** in solution is also red-shifted relative to those of **3** and pyrene, again owing to the π-extension. The emission of pyrene at 395 nm in solution is considered to result from the monomer form because of the low concentration, since pyrene is known to generate excimer emission at 480 nm at high concentrations (>10^−5^ M) [26–27]. The emission of **1** at 422 nm is also assigned to monomer emission. Compound **1** did not generate excimer emission in solution concentrations over the range of 10^−4^–10^−7^ M and, due to the poor solubility of this compound, spectra at concentrations above 10^−4^ M could not be acquired. The emissions of **1** and pyrene in the solid state (at 473 and 463 nm) were red-shifted relative to those observed for these compounds in solution. The red-shifted emission of pyrene in the solid state originates from the excimer state of the crystal [26–27]. In the herringbone packing of **1**, the distance associated with the CH–π interaction is 3.0 Å which is sufficiently close to form an excimer (Figure 2f). The angle of the pyrene moieties resulting from the CH–π interaction is approximately 40° (Figure 2f), which enables the partial π–π interactions. Judging from the crystal features of **1**, the red-shifted emission of **1** in the crystal is also assigned to the excimer state. The quantum yields of pyrene in solution and in the solid state are almost equal (0.64 and 0.68). In contrast, the quantum yield of **1** in the solid state (0.10) is decreased significantly from that in solution (0.82). This exciton quenching may be due to the effect of the sumanene moiety, since the quantum yield of **3** is low both in solution and in the solid state.

**Table 1:** Absorption, emission and quantum yield data for **1, 3** and pyrene.

compound	λ_abs_ (nm)^a^ (ε = 1 x 10^5^ , mol^–1^cm^–1^L)	λ _em_(nm) ^b^	Φ^c^

**1** (solution)	243 (0.98), 280 (0.98), 355 (0.57)	422	0.82
**3** (solution)	278 (0.80)	375	0.02
pyrene (solution)	242 (1.01), 274 (0.64), 337 (0.61)	395	0.64^d^
**1** (solid)	–	473	0.10
**3** (solid)	–	395	0.03
pyrene (solid)	–	463	0.68^d^

^a^Absorption spectra in CH_2_Cl_2_ (1.0 × 10^−5^ M); ^b^emission spectra in CH_2_Cl_2_ (1.0 × 10^−5^ M) or in solid state. Excitation at 280 nm for **1** (solution) and **3** (solution and solid), 300 nm for **1** (solid), 270 nm for pyrene (solution and solid); ^c^relative quantum yield in cyclohexane solution (5.0 × 10^−7^ M) with 9,10-diphenylanthracene as a standard or absolute quantum yield in solid state; ^d^reported quantum yield of pyrene in [26–27].

## Conclusion

The ability to predict the crystal packing of organic molecules is important in the design of functional organic compounds but remains challenging. In this regard, the columnar structure of buckybowl crystals resulting from convex–concave intermolecular π–π interactions is expected to be quite predictable and to serve as a directing force to provide specific crystal structures [16–17]. The present study demonstrates the promising possibility of utilizing the sumanene moiety as a directing group to obtain specific crystal structures.

## Experimental

### General

UV–visible absorption spectra were recorded on a JASCO V-670 spectrometer. Fluorescence spectra were recorded on a JASCO FP6500 spectrometer. Melting points were determined on a Standford Research Systems MPA 100 and were uncorrected. Infrared (IR) spectra were recorded on a JASCO FT IR-4100 spectrometer. ^1^H and ^13^C NMR spectra were measured on a JEOL JNM-ECS (Delta V5.0) 400 spectrometer at 23 °C at 400 MHz and 100 MHz. CDCl_3_ was used as a solvent and the residual solvent peaks were used as an internal standard (^1^H NMR: CDCl_3_ 7.24 ppm; ^13^C NMR: CDCl_3_ 77.00 ppm). Elemental analyses were measured on a J-Science Micro corder JM10. Mass spectra were measured on a JEOL JMS-777V spectrometer using electron impact mode (EI). Gel-permeation chromatography (GPC) was performed on JAIGEL 1H and 2H using a JAI Recycling Preparative HPLC LC-908W with CHCl_3_ as eluent. TLC analysis was performed using Merck silica gel 60 F254. All reagents and solvents were commercially purchased from Kanto, Wako, Nacalai, and Kishida and further purified according to the standard methods, if necessary.

#### Synthesis of **2**

Sumanene (**3**) (100 mg, 0.378 mmol), 6,6’-diiodo-2,2’-dimethoxy-1,1’-binaphthol (DIH) (144 mg, 0.378 mmol) and scandium(III) triflate (9.3 mg, 0.0189 mmol) were placed in a 50 mL dry flask under an Ar atmosphere. Dry CH_2_Cl_2_ (37 mL) was then added. The reaction mixture was allowed to stir for 2.5 h at rt. The completion of reaction was monitored by TLC (100% cyclohexane). The reaction was quenched by saturated aq. Na_2_S_2_O_3_ and the mixture was extracted with CH_2_Cl_2_ (50 mL × 3). The combined organic extracts were washed with water, brine, dried over Na_2_SO_4_, filtered through Celite, and evaporated. The residue was purified by GPC to afford pure **2** (106 mg, 80%) with recovery of **3** (10.0 mg).

#### Synthesis of **1**

Iodosumanene (**2**) (10.0 mg, 0.025 mmol), pyreneboronic acid (7.8 mg, 0.038 mmol) and palladium(II) acetate (1.2 mg, 0.0051 mmol) were placed in a 50 mL dry test-tube. Dry acetone (8 mL) and water (4 mL) was then added. The reaction mixture was allowed to stir for 12 h at 40 °C. The completion of reaction was monitored by TLC (100% cyclohexane). The reaction was diluted by CH_2_Cl_2_ and the mixture was extracted with CH_2_Cl_2_ (50 mL × 3). The combined organic extracts were washed with water, brine, dried over Na_2_SO_4_, filtered through Celite, and evaporated. The residue was purified by GPC to afford pure **1** (10.0 mg, 84%).

### Characterization data

#### Pyrenylsumanene (**1**)

Mp: 255 °C; IR (KBr) ν: 3039, 2895, 2780, 1396, 842, 788, 725, 683, 602, 488, 418 cm^−1^; ^1^H NMR (CDCl_3_) δ 8.68 (s, 1H), 8.20–7.99 (m, 7H), 7.71 (s, 1H), 7.40 (s, 1H) 7.19–6.90 (m, 4H), 4.84 (d, *J* = 19.6 Hz, 1H), 4.74 (d, *J* = 19.6 Hz, 1H), 4.53 (d, *J* = 19.6 Hz, 1H), 3.61 (d, *J* = 19.6 Hz, 1H), 3.45 (d, *J* = 19.6 Hz, 1H), 2.94 (d, *J* = 19.6 Hz, 1H) ppm; ^13^C NMR (CDCl_3_) δ 149.22, 149.21, 149.13, 149.12, 149.02, 149.10, 148.95, 148.94, 148.93, 148.73, 148,30, 148.18, 136.84, 131.62, 131.19, 130.95, 130.73, 127.71, 127.70, 127.69, 127.52, 127.51, 127.44, 126.09, 125.86, 125.77, 125.19, 125.09, 124.97, 124.67, 123.52, 123.40, 123.39, 123.25, 42.05, 41.99, 41.90 ppm; anal. calcd for C_37_H_20_: C, 95.66; H, 4.34; found: C, 95.38; H, 4.40; HRMS (EI) *m/z* calcd for C_37_H_20_ [M^+^]: 464.1565; found: 464.1570.

Crystallographic data have been deposited with Cambridge Crystallographic Data Centre: Deposition number CCDC-986895.Copies of the data can be obtained free of charge via http://www.ccdc.cam.ac.uk/conts/retrieving.html.

## Supporting Information

File 1 CIF file for the pyrenylsumanene crystal.
